# Pathological Researches on Phthisis

**Published:** 1836-01

**Authors:** 


### PART SECOND.

### Bibliographical Notices.

---

Art. I.—*Pathological Researches on Phthisis.* By E. Ch. Louis, m.d., Physician to the Hospital of La Pitié, &c. *Translated from the French, with Introduction, Notes, Additions, and an Essay on Treatment,* by Charles Cowan, m.d., &c.—*London,* 1835. 8vo. pp. li. 388.

The original work, of which we have here a translation, having been ten years before the tribunal of the medical public, and that tribunal having long returned an almost unanimous verdict in its favour, it is altogether unnecessary for us to enter into any details respecting either the character of the book itself, or the subject of which it treats. We cannot, however, omit any fair opportunity of bearing our individual testimony to the very extraordinary merits, both of the work and of its author. Regarded as a record of the *pathology* of phthisis, when formally developed, it is not only the best and completest that exists, but it is the very model of what a work on such a subject should be. The facts are sufficiently numerous, well selected, well arranged, detailed with great clearness, and all bearing the sovereign impress of truth; and not only of truth in the main, but of pure, unalloyed, exclusive truth. It is the crowning glory of M. Louis,—and this is a glory which would illustrate his name in any country, but which renders it still more illustrious in his own at the present time, where imperfect views and opinions are, by the lively imagination of the propounders, so readily converted into facts, from which the most momentous conclusions are, with equal inconsequence and rapidity, deduced:—it is, we say, the crowning and distinguishing glory of M. Louis, that he follows the only true path in physic, the Baconian path of rigid observation; departing no further from it than seems warranted by the progress he has made, and the knowledge to which this has led him. The consequence has been such as all who are acquainted with this method of philosophizing might expect, the production of the volume before us, which, whatever revolutions medicine as a science may undergo, must for ever remain a record of most important facts, available alike to practical men and theorists, and destined to perpetuate his name with the small number who have really added to the enduring materials of medical science.

In speaking thus of the present work of M. Louis, we must, however, be understood as not extending our commendations beyond the qualifications as given above. It is only as a picture of the full-formed disease,—it is only as a specimen of pure pathology and semeiology,—it is only, in short, as an elaborate and complete natural history of phthisis, from its formal development to its final close, that we commend the treatise before us. For the exposition of the remote causes of phthisis, —for the discrimination of the earliest indications of its invasion,—for the detection (if we may be permitted the expression) of the microscopic ova of the larva which, when developed, is destined, an envious kanker, to destroy the fair rosebud in which it is deposited,—we look in vain to the pages of our author; and, in the work as originally published, the all-important subject of treatment was entirely omitted. With these defects, we cannot, of course, recommend the treatise of M. Louis as a complete monograph on the subject of consumption; but, such as it is, and to the extent to which it goes, we do most earnestly, as we can most conscientiously, recommend it, as a production of first-rate excellence, and indispensable to every one who is desirous—and who that practises physic is not desirous?—of thoroughly understanding the nature, and habitudes, and indications of the most common and most fatal of diseases.

We consider the members of the profession in this country under great obligations to Dr. Cowan for the present which he has made to them of M. Louis's work in an English dress. If it is extraordinary that the translation has been delayed until the present time, the eagerness with which it is now hailed by the profession is a sufficient proof of the intrinsic value of the original, and that it is, as we have already said, above the mere accidents of time; and we are happy to think that the work has fortunately fallen into the hands of one well qualified, both by his medical knowledge and his literary acquirements, to do it justice. It is evident, from the numerous and important additions which Dr. Cowan has made to the original, as well as from the general character of his translation, and the clear and philosophical views announced in his excellent preface, that Dr. Cowan was well qualified to undertake the important task which he has here executed, and that the laborious duty of translation has been dignified in his hands. In future years, (for we presume that Dr. Cowan is still young,) no doubt the profession will have to thank him for something exclusively his own. In the mean time we think he has shewn much more, both of judgment and taste, in enabling all his countrymen to render themselves familiar with the riches of one of the greatest of their foreign contemporaries, than if he had sought to acquire a precocious and fleeting notoriety, by any immature original speculations.

We have read considerable portions of Dr. Cowan's version, and have compared it with the original; and, as we can speak confidently and conscientiously both of its fidelity as a translation and of its general correctness as a composition in English, we recommend it in the strongest terms to all of our readers who are unacquainted with the original treatise. To be sure, Dr. Cowan's translation is by no means free from faults, and we noted, as we run over its pages, several expressions not very accurate and not very English: still, on the whole, we are much pleased with it, and are bound to say of it, what we fear cannot be predicated of all medical translations, that it is truly "done into English," according to the good honest phrase of the old time. As little matters of a general and intentional kind to which we object in Dr. Cowan's version, we may name the employment, in the English, of the term *observation* for the same word in the original: *case,* we humbly conceive, ought to have been substituted for the French word, as the word *observation* is otherwise appropriated in medical language. We make the same objection to the use of the word *sectio,* uniformly employed by Dr. Cowan as synonymous with what is commonly, though barbarously, termed in English medical writings, *post-mortem examination,* and which stands for the much more proper expression *ouverture du cadavre,* uniformly employed in the original. Objecting as we do to Dr. Cowan's term, we must admit that, though less definite, and therefore, philosophically speaking, less proper than the one quoted above as the more common one in English medical writings, it is decidedly less offensive to our eyes and ears than that intolerable barbarism; and we verily think the man who shall invent and successfully introduce a word, term, or phrase, equally significative with that un-English, un-Latin adjective or substantive, (which shall we call it?)* *post-mortem,* will deserve the thanks of all succeeding medical authors and medical reviewers.

Another general peculiarity in Dr. Cowan's translation is the niggardly use of the definite article *the* in the history of the cases, and of the cadaveric examinations; a slight fault, indeed, but which forces itself on the reader's notice, from the frequency of its occurrence: "adhered to costal pleura," "bile in gall-bladder," "membrane of trachea," and so on. Probably, Dr. Cowan adopted this mode of expression for the sake of brevity; but the space and time are purchased at too dear a price. An occasional mistake, also, evidently arising from heedlessness and hurry, met our eye here and there; but they are in very small number, and are only conspicuous because of the general goodness of the context.

We cannot conclude this brief notice of Dr. Cowan's translation without adverting once more to what is his own more immediate performance, the preface to the treatise: and we do this for two reasons,—first, because we wish to lay before our readers an interesting fragment of what may be truly called the professional or intellectual history of M. Louis; and, secondly, because we cannot have a better opportunity of calling the attention of our younger professional brethren to the particular method, or instrument, with which this author has worked out all that he has accomplished in medicine. This instrument is simply the rigid registration of all the facts that present themselves in the observation of cases of disease, and the classification of these facts in such wise as fairly to elicit the general results. From the form and mode of registering the phenomena, and calculating the results, the method has been styled "the numerical method;" and, although, as Dr. Cowan justly remarks, M. Louis has no pretensions to be its discoverer, inasmuch as it has been followed, more or less, by all the more accurate observers in all the natural sciences, still "he is fairly entitled to the merit of having been the first who has rigorously and extensively applied

* We are not a little surprised to find Dr. Cowan, whom we had given the credit of adopting the word sectio, objectionable as it is, out of mere dislike to the barbarism *post-mortem,* when used adjectively, actually employing the same word as a *substantive,* which is even a more intolerable nuisance than the other: thus, "he followed the visits, *post-mortems,* and lectures, &c." Note, p. xvi.

it to medicine;" or, perhaps, as we should have said, "the individual who has most rigorously and most extensively applied it."

In the following extracts, the peculiarities and merits of this method are well exposed, and the progress and proceedings of M. Louis are given with only their true colouring. The whole details are extremely creditable to Dr. Cowan; and, with the strong conviction which we have of their importance to all who are now entering on the most important part of *the study* of their profession, *the practice* of it, we make no apology for their length. The numerical method requires for its successful application nothing but what every well-educated physician and surgeon may possess, and ought to possess, namely, industry and patience in observing, and fidelity in recording facts, and common attention and caution in arranging the necessary results. It needs neither a piercing intuition nor a quick fancy, neither a capacious memory nor profound reasoning powers; yet it is capable of effecting greater things in medicine than any of these,—we had almost said, than all of these,—without it. In our next Number, [see a Review of M. Louis's work on the Effects of Bleeding, &c.,] will be found many of M. Louis's own remarks on this method. Still, we think the following account of it by Dr. Cowan may obviate certain objections that would be very likely to rise up in the mind of the English reader to the adoption of what may probably be considered as only an ingenious novelty.

"Our author presents an interesting example of the effect produced upon the mind by the contemplation of the uncertain nature of much of our medical knowledge; and he is also an illustrious proof of what the exertions of a single individual can effect, when, unfettered by theory or system, they are steadily directed to the simple, unbiassed observation of facts. M. Louis, from the age of seventeen to thirty-three, studied and practised medicine in Russia, with considerable success. Gifted with a naturally active and enquiring mind, the multitude of opinions, contrasted with the paucity of facts, could not fail to create great dissatisfaction and uncertainty as to the validity of many of the principles most generally admitted, and on which much of our practice was founded.

"Accidental circumstances, at the close of this period, bringing him to Paris, he soon became acquainted with, and eagerly studied, the writings of the celebrated Broussais; at the same time assiduously following that distinguished pathologist, both in the hospital and lecture-room. The impression produced upon his mind by this direction of his studies was, that, while M. Broussais evidently proved others to be wrong, he was very far from demonstrating himself to be right; that, while he rendered palpable the doubts which might reasonably be entertained respecting many of our present principles, he had failed to substitute any thing more satisfactory in their place. From this moment M. L. resolved to devote himself *exclusively* to observation, solely actuated by a desire to relieve oppressive doubt and uncertainty, and with no intention of ever giving publicity to his labours. He at once decided on remaining at Paris, as affording the best opportunities for prosecuting his intentions, and entered the hospital of La Charité as a *clinical clerk*, under his friend Professor Chomel. For nearly *seven years*, including the flower of his bodily and mental powers, (from the age of thirty-three to forty,) he consecrated the whole of his time and talents *to rigorous impartial observation.* All private practice was relinquished, and he allowed no considerations of personal emolument to interfere with the resolution he had formed. For some time his extreme minuteness of enquiry and accuracy of description were the subjects of sneering and ridicule, and *cui bono* was not unfrequently and tauntingly asked. The absence of any immediate result seemed for a time to justify their contempt of a method involving too much labour and personal sacrifice to be generally popular or easily imitated, and M. Louis himself at moments almost yielded to the increasing difficulties of the task he had undertaken. No sooner, however, were his facts sufficiently numerous to admit of numerical analysis, than all doubt and hesitation were dissipated, and the conviction that the path he was pursuing could alone conduct him to the discovery of truth, became the animating motive for future perseverance. Many of the results to which he arrived soon attracted general attention, and, among those who had formerly derided his method while they admired his zeal, he found many to applaud, and a few to imitate. From this moment may be dated the presence of that strong impression of the necessity of exact observation, by which the school of Paris has been since so distinguished, and which is now gradually pervading the medical institutions of the continent and our own country. It is undoubtedly to the author of the present volume that we ought to ascribe the practical revival of that system which had for ages been verbally recognised, but never before rigorously exemplified. For the last five years he has been physician to the hospital of La Pitié: the number of advanced students (principally English, American, and German,) who follow his visits and clinical lectures, are the best testimonies to the indefatigable zeal and talent with which he still pursues his investigations, and, contrasted with the now-deserted wards of M. Broussais, forms a practical illustration of the striking change which has been effected in the spirit of medical enquiry.

"With no preconceived views of his own to establish, (and we believe no one who *has* will observe seven years!) all results from such researches cannot fail to address themselves to our confidence; and, in the present instance, they have not only the additional value of having been made at a period of life when the judgment is matured and fancy regulated, but by one who, so to speak, *began his studies* after several years' practical experience of their difficulties. He regarded each individual example of disease as a problem which could only be solved by patient and exact observation: with this conviction, he studied *all* the functions during life, from the commencement of the disease to its termination: for the same reason he examined all the organs after death; and, when attempting to arrive at any general conclusion, he not only analysed the facts he had collected relative to that disease, but submitted them to a rigorous comparison with other diseases which were at all analogous. It is evidently one thing to determine the series of symptoms, or alterations of structure, which are present in any particular affection, and another to discover what symptoms or alterations are special and characteristic: the one is obtained by confining ourselves to the disease itself, the other can alone result from comparison. A very short time was sufficient to make the discovery that *observation* was immensely difficult; a fact which authors have hitherto overlooked, thus plainly proving that they themselves observed incompletely. The power of correct observation is not the attribute of ignorance, but is, *cæteris paribus*, always proportioned to the knowledge the individual possesses. With what additional profit and success does the painter, the sculptor, the naturalist, observe, after a long cultivation of their respective arts, and how numerous are the details detected, which would wholly escape the unpractised novice? Now, if an accurate conception of external characters, when passive under the eye of the observer, demands long and patient exercise for its acquirement, how much greater must be the difficulties surrounding the complicated machine of the human figure, under all the varied influences and the innumerable modifications of which it is susceptible? The phenomena are not only complex and ever varying, but they must often be examined through the distorting medium of a suffering and fanciful mind, and are frequently described with the intention to mislead and deceive.

"Not to be continually the dupe of such sources of fallacy, (and the most practised do not always escape,) requires long habit and extensive general knowledge, and no one can have APPRENTICED himself, as the author in his preface remarks, to the *trade* of minute and rigorous examination, without a deep conviction of the difficulties attending it, and the necessity of long continued perseverance." (*Pref.* p. xviii.—xxi.) * * *

"But observation, however extended and exact, is of itself insufficient to generate conclusions; for, collected as our facts must have been, through a series of months or years, and consisting of an infinite variety of details, no memory could recall, and no mind could grasp, their complicated relations with each other. To accomplish this, the '*numerical method*' is necessary; that is, *counting* the number of all the individual facts, comparing their relative frequency in cases of a particular class, and then determining their real value by a comparison with facts of other classes, which have also been reduced to similar elements. This is the plan pursued by our author, and which must be adopted by all who would seek to establish truth and arrive at general results. Hitherto we have satisfied ourselves with the authority of *experience;* and its currency in medicine is such, that any distinct definition of its value has scarcely been attempted. But let us enquire what is really included by experience? Is it not the expression of the conclusions of the mind upon one or more subjects, to which the attention has been habitually directed? Is it not, simply, the final impression produced by a review of the past? If the discovery of truth be its tendency, why has individual experience been hitherto so discordant? The answer is easy. In a science like medicine, where the difficulties of observation are so great, and the objects to be observed so numerous; where theories bias, and individual peculiarities necessarily exert their influence, nearly all, if not all, the conclusions of mere experience are varying and fallacious. Who does not feel himself naturally inclined to study one class of affections more than another, to be arrested by particular symptoms, to be more interested with facts which apparently coincide with some favourite views he has either adopted from others, or insensibly formed during the course of his studies? How strongly all *extraordinary* facts, and what we call *interesting cases*, are engraven upon the mind, and for ever prominent in the retrospect, while the great mass of *ordinary*, and consequently *important*, occurrences are overlooked or forgotten? Some unhoped-for success attending the means we employ, how firmly has it associated the cure of the disease with the specific nature of the remedy; and how easily do we admit as a fact what the observation of another proves to be the mere expression of a coincidence? Every practitioner has his peculiar therapeutics, his favourite dogmas to support, and successes to boast; and, when we reflect on the innumerable opinions which exist on all complicated subjects, where conclusions are founded on the materials of unrecorded individual experience; materials which opportunity, education, and a thousand accidental circumstances, are for ever modifying; we cannot, I think, be surprised that the results of experience in medicine have not been more uniform and satisfactory.

"While anxious to impress upon the reader our conviction that unrecorded experience can never become the corner-stone of any science whatever, we admit that it has justly acquired, in a few rare instances, unusual relative value, from the capacious intellect and retentive memory of some highly favoured minds. Devoted, as we have described our author to have been, to the observation of facts, and divested, as he was, from the very state of mind which actuated him to the course he so undeviatingly pursued, from all *preconceived opinions*, yet it was impossible that, during so long a period of time, his mind should not have been unequally impressed by the phenomena before him, and have unknowingly fixed some in its remembrance, to the exclusion of others, instinctively allotting them a relative value, and arranging them to favour some *a priori* conclusions. Now, no circumstances could possibly have been more favourable to test the value of experience than those in which M. Louis was placed; yet when, at the close of his labours, he submitted all his facts to the unerring test of arithmetical analysis, *in every instance were the* a priori *conclusions, which he had formed from the recollection of his own facts, found to be erroneous.* This most remarkable result ought to be indelibly engraven on the mind of every observer, and inspire a doubt as to the validity, not only of the experience of others but of what he has hitherto perhaps considered almost infallible, *his own.*" (*Pref.* xxii.—xxiv.)

"The numerical analysis requires, in the first place, a sufficient number of carefully collected facts on the same subject, our object is then to classify their corresponding elements, so that not only are all the details of those facts successively submitted to the mind, but their relative frequency and value more easily estimated. To effect this, synoptical tables are indispensable, and their number necessarily proportionate to the complex nature of the facts we are analysing. Each organ, for instance, must have a separate column, which includes its description in every case we intend to make use of, adopting as near as possible similar terms for similar conditions.

"This, however, alone would be very inefficient, as in a complicated structure like the lungs, where so many alterations may occur, a long series of minute descriptions would defy analysis from simple inspection; each organ therefore, in its turn, becomes the subject of a separate table, which also consists of subdivisions proportionably numerous as the object we examine is simple or complex. When we have thus arranged all the elements of our facts, we compare the results of our different columns with each other, having it thus in our power to view them in their various relations, while we may at pleasure refer particular facts to their respective observations, the same number accompanying all the details which are scattered through a variety of tables.

"It will be remembered there is nothing arbitrary in this mode of proceeding, nothing left to individual caprice or preconception; for, in the arrangement of our tables, we perform a purely mechanical operation, indiscriminately putting down all the facts in their respective columns, without any reference to the conclusions to which they ultimately tend. The correctness, then, of any opinions we may form, is confirmed or rejected by a test over which we have no control, and the evidence of which no well-regulated mind can resist, while not only the relative importance of many facts to which our attention had been less distinctly directed, or which we had wholly forgotten, is forced upon our consideration, but we are also led to the discovery of what we have only casually or incompletely described.

"It will at once be perceived that certain laws require, for their elucidation, a much larger number of examples than others: where a hundred observations may in one case be sufficient, three times that number may be required under other circumstances. Indeed, as a general rule, the more complicated the objects we examine, the greater the number of facts necessary to establish our conclusions; for the same elements not being repeated in all, their relative aggregate number must vary, and their real value can only be estimated by tracing them through a larger number of analogous instances. Were we, for example, analysing one hundred cases of pleurisy, the value of any symptom invariably observed would be considerable, and perhaps sufficiently established; but, were it only present twenty times out of that hundred, its real importance would be much less positive, and require an additional number of facts for its determination.

"For the appreciation of treatment, the necessity for numerous facts is peculiarly apparent, for though a hundred cases would be valuable evidence in favour of any one system of cure, it is only by comparison with others that its real efficacy can be decided. There are also other sources of fallacy which must not be overlooked; such as the severity of the disease, the age and sex of the patient, the state of health at the time, the natural duration of the affection, the epidemic influences which may be present, &c.; these are all questions to be solved before we can arrive at any positive results. From these rapid reflections, we may form some idea of the numerous difficulties which surround every question of therapeutics, and feel the necessity of exercising the greatest caution in ascribing any definite value to a remedy before we have well determined, by numerously analysed facts, the exact circumstances under which its action has proved to be beneficial. No part of medical knowledge is more in want of some rigorous method of investigation than that of therapeutics, and this must ever be the case, until a system analogous to the one we have briefly described shall be generally adopted.

"It is not our intention, in advocating the numerical method, to conceal for a moment its difficulties: these are great and numerous, but at the same time they can never form any solid argument against its utility, though they will necessarily curtail the number of its disciples. It is, in fact, the only method in our power to pursue; it is the only control we can possess over assertion, the only test for opinion, and though not all we can wish, and no doubt will ever be found inadequate for the decision of many questions, yet its application to a sufficient number of facts must inevitably give us the most exact and best possible knowledge of those facts, and we would ask the individual who believes that science is founded upon facts, what more he would require?

"How could we have ascertained that tubercles in any organ of the body, after the age of fifteen, involved their presence in the lungs? That phthisis almost invariably commences in the upper lobes? That it is more frequent in women than in men? That pneumonia is more easily resolved in a tuberculated than in a healthy lung? That simple bronchitis commences at the base of the lungs, pursuing a course inverse to that of phthisis? That chronic peritonitis indicates pulmonary tubercles? That acute affections, when free from complication, are generally confined to one side of the body, or one part of an organ if single? How could these, and many other results, be obtained but by rigorous observation and numerical analysis? And what theory have we ever heard of, which could have led us to the same conclusions? Had they been advanced as the fruits of speculation, how absurd some of them would have appeared, and their very announcement would have almost ensured their rejection; but founded as they are on the evidence of facts, our ignorance of the laws on which they depend is no bar to their practical utility. We know of no considerations more directly in support of the numerical method, or more enc uraging to all who have the necessary opportunity and perseverance for its adoption, than this almost spontaneous creation of laws, which must have escaped the sagacity of reasoning, from the simple fact that, when demonstrated, they refuse to coalesce with any of our preconceived opinions." (*Pref.* xxv.—ix.)

We ought to have stated before, that the translator has added many valuable notes to different parts of the text; and that the last two chapters of the work, *on the Causes and Treatment of Phthisis*, which are extremely scanty and unsatisfactory in the original, have been greatly enlarged, almost indeed supplied by him. In these various additions, the translator has throughout evinced a sound judgment, and displayed an extensive acquaintance with the literature of his profession. He has proved himself fitted for the important task he has undertaken, by shewing that he possesses no mean share of that talent for observation, that acquired knowledge, that philosophical spirit, that industry and rigid honesty, which so happily distinguish the illustrious author of the original treatise.

